# An *in vitro* evaluation of the fatigue behavior of resin composite materials as part of a translational research cycle

**DOI:** 10.1016/j.dental.2024.06.010

**Published:** 2024-06-25

**Authors:** L.A.M.J. Crins, N.J.M. Opdam, M.C.D.N.J.M. Huysmans, Y. Zhang, B.A.C. Loomans

**Affiliations:** a Radboud University Medical Center, Department of Dentistry, P.O. Box 9101, Nijmegen 6525 EX, the Netherlands; b Department of Preventive & Restorative Sciences, School of Dental Medicine, University of Pennsylvania, 109 Levy Buidling, Philadelphia, USA

**Keywords:** Composite resins, Sliding contact, CAD/CAM, Wear rate, Fracture resistance, Fatigue resistance

## Abstract

**Purpose:**

This study aimed to reproduce and translate clinical presentations in an *in vitro* set-up and evaluate laboratory outcomes of mechanical properties (flexural strength, fatigue resistance, wear resistance) and link them to the clinical outcomes of the employed materials in the Radboud Tooth Wear Project (RTWP).

**Materials and methods:**

Four dental resin composites were selected. 30 discs (Ø12.0 mm, 1.2 mm thick) were fabricated for each of Clearfil TM AP-X (AP), Filtek TM Supreme XTE (FS), Estenia TM C&B (ES), and Lava Ultimate (LU). Cyclic loading (200 N, 2 Hz frequency) was applied concentrically to 15 specimens per group with a spherical steatite indenter (r = 3.18 mm) in water in a contact-load-slide-liftoff motion (105 cycles). The wear scar was analysed using profilometry and the volume loss was digitally computed. Finally, all specimens were loaded (fatigued specimens with their worn surface loaded in tension) until fracture in a biaxial flexure apparatus. The differences in volume loss and flexural strength were determined using regression analysis.

**Results:**

Compared to AP and FS, ES and LU showed a significantly lower volume loss (p < 0.05). Non-fatigued ES specimens had a similar flexural strength compared to nonfatigued AP, while non-fatigued FS and LU specimens had a lower flexural strength (p < 0.001; 95 %CI: −80.0 – 51.8). The fatigue test resulted in a significant decrease of the flexural strength of ES specimens, only (p < 0.001; 95 %CI: −96.1 – −54.6).

**Clinical relevance:**

These outcomes concur with the outcomes of clinical studies on the longevity of these composites in patients with tooth wear. Therefore, the employed laboratory test seems to have the potential to test materials in a clinically relevant way.

## 1. Introduction

Tooth wear is a physiological process that, depending on the extent and rate of wear, may become pathological [1]. Especially in the context of less caries among younger people and a growing elderly population retaining their natural teeth, restorative treatment of tooth wear may be indicated more often. In case of a full rehabilitation of patients with severe tooth wear, minimally invasive restorative procedures are preferred that preserve the worn teeth and add restorative material without or with very limited preparation. This is a challenging restorative procedure as adhesive restorations in an increased vertical dimension of occlusion are required [1]. In those cases, the complete occlusal load is born by the restorative material and, as tooth wear patients are a high-risk group for parafunctional activities, these materials are challenged to their limits on fracture strength and wear resistance.

The Radboud Tooth Wear Project (RTWP) [2] focuses on the clinical management of patients with advanced stages of tooth wear. Within this project, multiple prospective clinical studies are embedded. A large group of patients (approximately 140) with generalized, moderate to severe, tooth wear was restoratively treated with direct and indirect composite materials as well as CAD/CAM composites [3–8]. The prospective studies evaluating direct composite restorations showed acceptable clinical success [3,5]. Whenever the clinical success was compromised, fracture of restoration was the main failure modality, with chip fractures occurring the most frequently within the observation period [3–5]. Although it was not a clinical concern within the observation time, wear of the materials could limit the survival of these composite restorations, on the long term. Comparing the wear for the two composite materials that were used in two prospective studies (hybrid and nanocomposite), no differences were observed [7,8]. A striking difference in clinical success was observed when directly comparing direct composite to conventional indirect composite on molar teeth [3]. In that randomized controlled trial, material fracture was again the main failure modality, and it was concluded that the indirect composite was not suitable for restoring worn molar teeth. However, similar failure rates for CAD/CAM indirect composite restorations to those for directly applied composite restorations were found in another prospective study. This suggests that the application technique itself is not necessarily causing higher risks for restoration failure. One of the factors in the poor clinical result of the conventional indirect composite restorations on molar teeth may be the individual susceptibility to fatigue or failure of the composite material. *In vitro* studies can help to understand specific clinical outcomes.

The classical approach to the evaluation of the performance of dental materials is to initially perform laboratory studies aiming to anticipate *in vivo* results prior to undertaking clinical trials. Research aimed at converting results in basic research into results that directly benefit humans is often called translational research and can be described as “from lab to clinic”. Unfortunately, *in vitro* tests struggle with their clinical relevance [9–11]. To improve the ‘clinical value’ of laboratory tests, information from the clinic should be incorporated into the laboratory set-up. The first possible step is to emulate clinical presentations in laboratory set-ups. When successfully emulated, modifications to laboratory set-ups may be introduced to allow for better preclinical testing of restorative materials and techniques that can benefit *in vivo* outcomes. These steps together can be repeated endlessly in a cycle best characterized as “from the clinic to the laboratory, and then back to the clinic”.

From the perspective of the RTWP a better understanding of the materials’ strength and wear resistance is needed. The clinical relevance of simple tests looking only at fracture strength or simulated wear, however, is not clear [9]. Fatigue testing is a time-consuming procedure but may be the most appropriate *in vitro* test for testing materials that are used for tooth wear rehabilitations[9,12,13]. Many *in vitro* methods for testing of dental materials use loading and wear simulation devices and present variations in chewing simulation mechanisms. Some devices apply mechanical cyclic compressive loads, with or without sliding. A method that has been shown to produce fatigue failures similar to those found clinically in tabletop ceramic restorations [14] applied mechanical compressive loads without sliding but did not show levels of (contact) wear. Static repetitive compressive loads may be assumed not suitable to reproduce the clinical presentation of restoration fracture and restoration wear as found in tooth wear patients. Other simulators, such as the Rub&Roll device [15], apply loads in a rolling motion in an endeavour to resemble chewing cycles. Recently, it was shown that the device was capable to produce deterioration compatible to erosive cup-shaped lesions [16] and to emulate the surface deterioration effects observed in composite restorations placed in patients with severe tooth wear [17]. Another recent study showed that repetitive impact-sliding movements allow for wear development and material fatigue at a lower number of cyclic loading [18] because it introduces superficial damage in some materials and it institutes friction between the indenter and substrate that intensifies both tensile and compressive stresses in the sliding action [19,20].

To better understand the materials’ effect on clinical performance of restorations this study aimed to reproduce and translate clinical presentations in an *in vitro* set-up and evaluate laboratory outcomes of mechanical properties (flexural strength, fatigue resistance, and wear resistance) and link them to the clinical outcomes for the employed materials.

## 2. Methods and materials

### 2.1. Materials

In this study, dental resin composites were used, for which also clinical results from the RTWP for the treatment of patients with moderate to severe tooth wear were available [3–8]. Two composites for direct application (Clearfil ^™^ AP-X, Kuraray (AP) and Filtek ^™^ Supreme XTE, 3 M (FS)) and two composites for indirect application (Estenia ^™^ C&B, Kuraray (ES) and Lava Ultimate, 3 M (LU)). Two subtypes of resin composite were included as AP and ES are micro-hybrid composites and FS and LU are nanocomposites. The used composite materials including abbreviations and compositions are listed in Table 1. A list of all materials and instruments used in this study can be found in the supplementary Table. The study included four composite materials that had both a control group and a test group. Only in the test group, a fatigue test was applied by mechanical cyclic loading. The number of specimens per group was set at 15.

### 2.2. Fabrication of specimens

#### 2.2.1. Direct resin composites

**Fig. 1.**
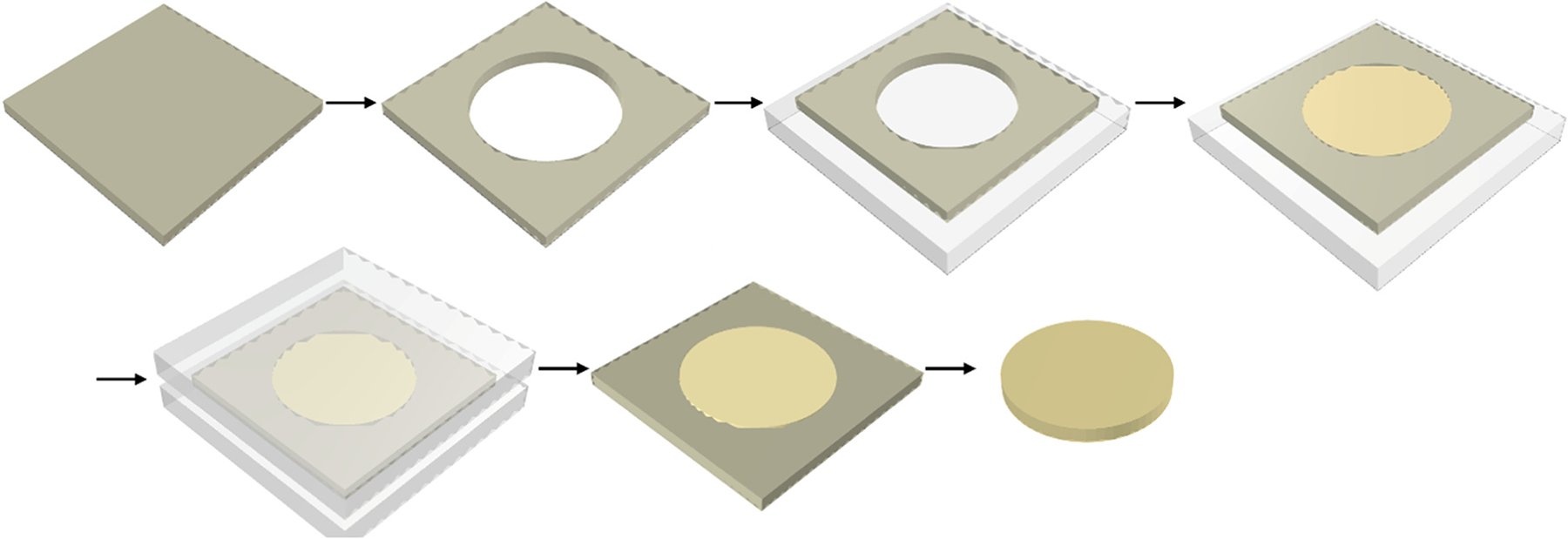
Schematic presentation of the production of direct resin composite discs.

A cylindrical defect with a diameter of 12.0 mm was created in a high-density polyethylene plate of 1.2 mm thickness, Fig. 1. This resulted in an individualized mould with dimensions of 12.0 by 1.2 mm (diameter x height). The mould was placed on a flat glass plate and the defect was compacted in bulk with AP and FS, respectively. Then, a second glass plate was placed on top and pressed onto the uncured composite. Light curing was performed through the glass plates using a polymerization unit (Bluephase 16i, Ivoclar, average output 1200 mW/cm^2^) for 20 s from the upper surface and 20 s from the lower surface. The manufacturing process resulted in disc-shaped specimens that were later manually polished using 800-grit wetted sandpaper on both sides to mimic clinical polishing with fine-grit diamond burs.

#### 2.2.2. Indirect composites

The specimens of ES were fabricated using the same mould and application and polishing procedure as the direct composites. The difference with the direct composite specimens was that ES specimens received an additional 270 s (310 s in total) of light curing from the upper surface and were also heat-cured in an oven (110° C, 15 min), according to the manufacturer’s instructions.

**Fig. 2.**
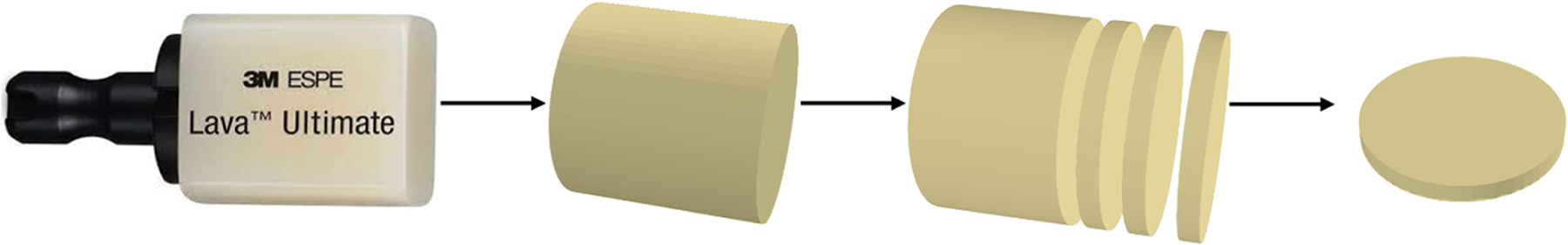
Schematic presentation of the production of composite discs out of prepolymerized blocks.

Pre-polymerized blocks of LAVA Ultimate (LU) were milled into cylinders with a diameter of 12.0 mm using a milling machine (CEREC Primemill, Dentsply Sirona). These cylinders were sectioned into discs with a thickness of 1.2 mm using a diamond blade with water-cooling in a universal cutting machine, Fig. 2. Polishing was performed identically to the other groups.

### 2.3. Fatigue test by cyclic loading

Specimens in the four test groups were cemented using Multilink^®^ Automix (Ivoclar) onto previously hydrated (30 days storage in distilled water [21]) fiber glass-reinforced epoxy resin (G10, Acculam, Yonkers), a dentin-analogous material [22]. G10 substrates were randomly chosen, and the adhesive surface was air-dried for 60 s. Then, Monobond Plus (Ivoclar) was applied to the surface with a microbrush and was left to react for 60 s. The surface was subsequently dried with a stream of air. The disc-shaped specimens were cleaned in an ultrasonic bath with ethanol for 5 min and then dried using an air stream. The adhesive surface was cleaned with 4.5 % hydrofluoric acid (IPS Ceramic Etching Gel, Ivoclar Vivadent) for 20 s, rinsed with air/water spray, and dried with air. A silane coupling agent (Monobond Plus, Ivoclar) was applied to the surface with a microbrush and left to react for 60 s. The specimens were cemented, using Multilink Automix (Ivoclar). Light curing of the luting agent was carried out by a LED curing light with an irradiance of 850 W/cm^2^ for 40 s for 4 consecutive times from different directions (Ultra Lume LED 5, Ultradent). After cementation, the specimen-G10 assemblies were stored in distilled water at 37 °C for 7 days for hydration. For reasons of standardization, specimens of the control group were also stored in distilled water at 37 °C for 7 days.

**Fig. 3.**
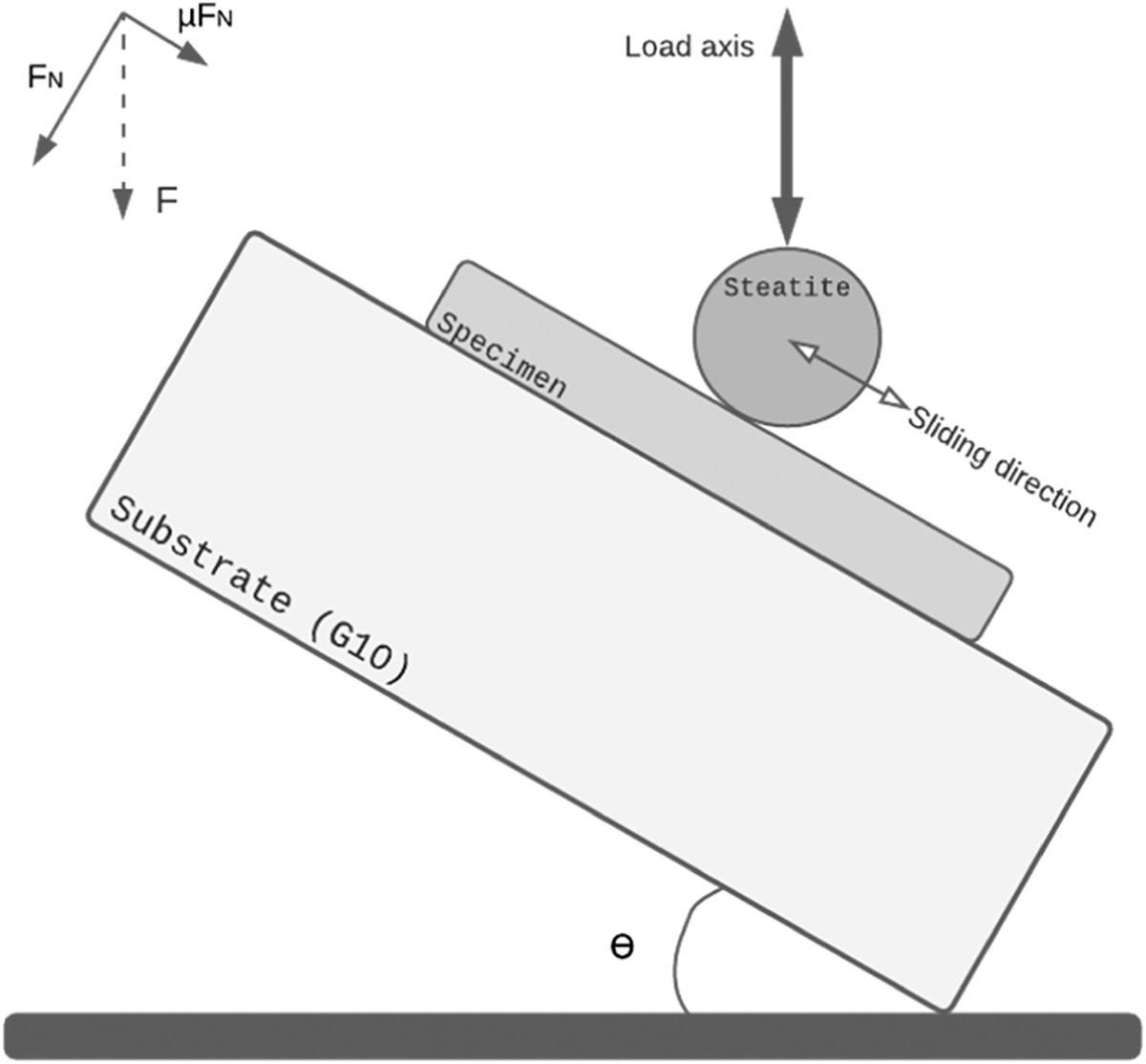
Schematic presentation of the fatigue loading configuration used in the study. The specimen was cemented onto a substrate and subjected to cyclic indentation with a spherical steatite indenter. A 30° inclination was used to facilitate a sliding motion of the indenter over the specimens’ surface. The indenter slides over the specimens’ surface for about 1 mm in the cyclic indentation.

The mechanical fatigue test was performed in a mouth-motion simulator (ELF-3300, EnduraTEC Division of TA Instruments). The specimens-G10 assemblies were placed onto an inclined block (θ = 30°) to generate off-axis loading of the indenter [19]. The vertical loading of a spherical steatite indenter (radius 3 mm) resulted in an initial contact of the indenter with the specimen’s surface at which the maximum load was transferred while sliding down (for about 1 mm). After the maximum load, which was set at 200 N, was reached, the load was reduced, and the indenter lifted off from the specimen’s surface while returning to its original position [23] The cycle can be described by contact-load-slide-and-back motion, as depicted in Fig. 3. Specimens were subjected to 10^5^ cycles of loading with a frequency of 2 Hz. The fatigue test was performed in distilled water.

### 2.4. Wear quantification

After the mechanical fatigue test, 7 specimens per group were inspected for wear using a non-contact Laser Abrasion measurement System (LAS-20, SD Mechatronik GmbH, Feldkirchen-Westerham, Germany) which does not require a stone replica or surface treatment of the specimen [24]. The resolution was set at 0.001 mm. The scanned data was saved as a point cloud in an STL file and transferred to Geomagic Wrap Software (v 2017). Within this software, noise and spikes in the point clouds were removed by the function ‘reduce noise’ and ‘remove spikes’. Additionally, small ‘holes’ in the point cloud were filled in by the software using the function ‘mesh doctor’. The next step was to introduce a plane (function plane shape) and position it parallel to the specimen’s unworn surface using the best-fit function. Subsequently, the edges of the wear scar were selected, and the polygon was trimmed to these edges. That resulted in the removal of the unworn surface adjacent to the wear scar. The selected edges of the wear scar were extruded (by 0.1 mm), using the function ‘extruded boundary’, with the goal to create a ‘watertight’ 3D volume. Then, this 3D volume and the reference plane were combined in which the reference plane acted as a cut-off. With the function ‘Boolean; subtract’, a 3D volume between the reference plane and the scanned surface of the wear scar could be realized. With the software function ‘compute volume’ the volume (μm^3^) of the wear scar was assessed.

### 2.5. Flexural strength tests

After being subjected to the mechanical fatigue test, and for some specimens also wear quantification, the G10 substrate of specimen-G10 assemblies was carefully cut, leaving a thin layer of G10 attached to the composite disc. The residual G10 was then removed by polishing the cementation surface of specimens using wetted sandpaper (grit 800). After polishing, the thickness of the composite specimens was measured.

**Fig. 4.**
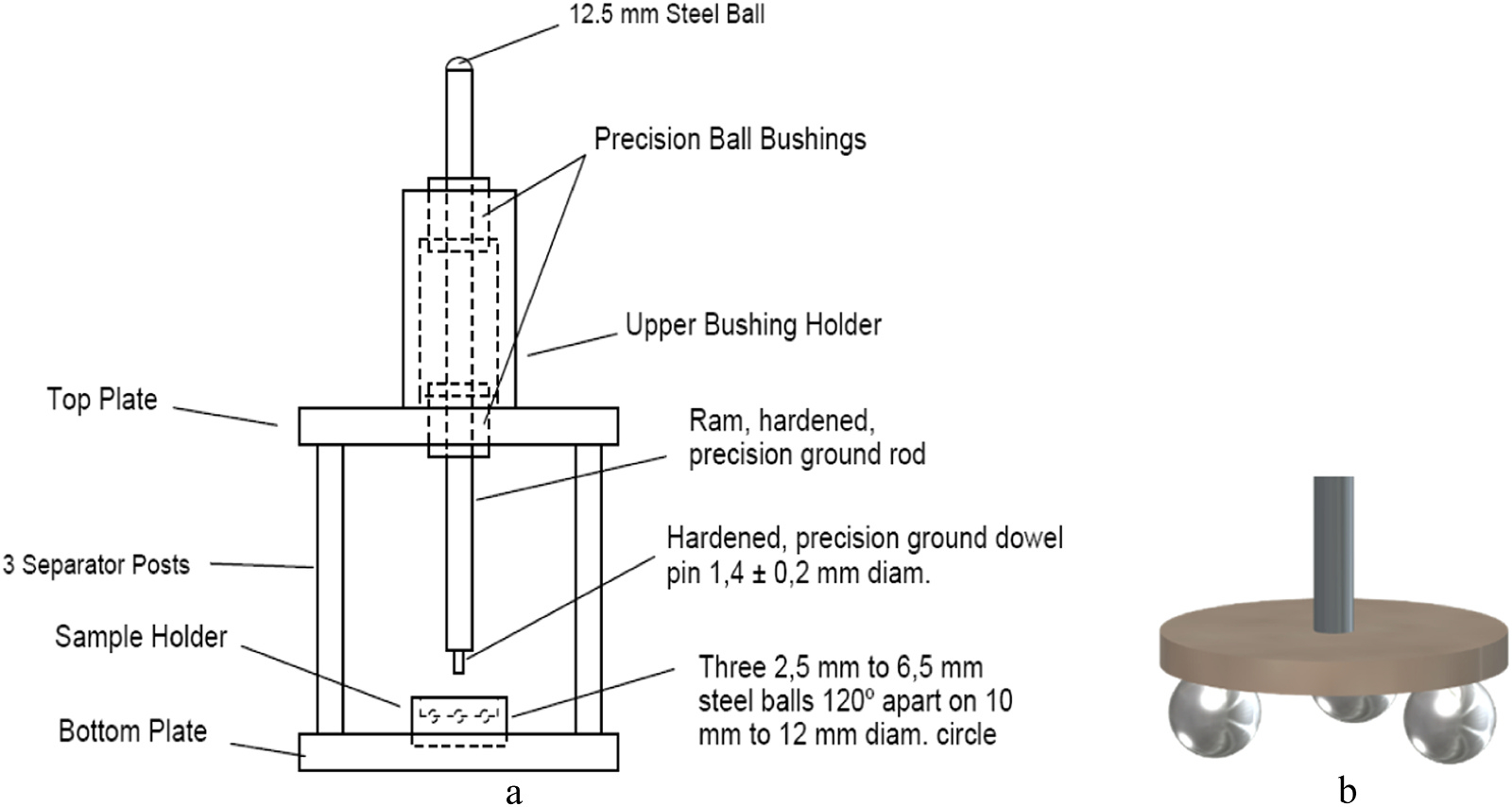
a: Schematic of a fixture for the piston-on-three-ball test. b: Schematic of a composite disc in the P3B-test.

A universal mechanical testing machine (model 5566; Instron, Norwood MA, USA), using a load cell capable of measuring applied loads of between 10 N and 1000 N ( ± 0.1 N), was used. The crosshead speed was set at 0.5 mm/min. A software program (Bluehill LE for Basic Testing, Instron) controlled the testing machine. Specimens were mounted in a piston-on-three-balls set-up (P3B-test) (ISO:6872), Fig. 4a. Three hardened steel balls with a diameter of 3.17 mm, were positioned 120° apart on a support circle with a diameter of 8.0 mm. Specimens were placed concentrically on these support balls with fatigued specimens having the wear facet on the flexural side [25]. The load was applied with a flat punch with a diameter of 1.37 mm at the center of the specimen, Fig. 4b. All specimens were subjected to a single load to failure test. The maximum load at the moment of fracture was noted.

### 2.6. Statistical analysis

Residuals in the data were checked. The volume of the wear scar after completion of the fatigue test was analysed in a regression analysis with the material as a variable. The differences in flexural strength were analysed using a regression analysis with both the material and the fatigue test as variables. Also, the interaction between the material and the fatigue test was checked in the regression analysis. Differences between materials were displayed in graphs showing the 95 %CI of the mean values of both wear volume and flexural strength. R-software (v 4.1.3) was used to perform the analyses.

## 3. Results

**Fig. 5.**
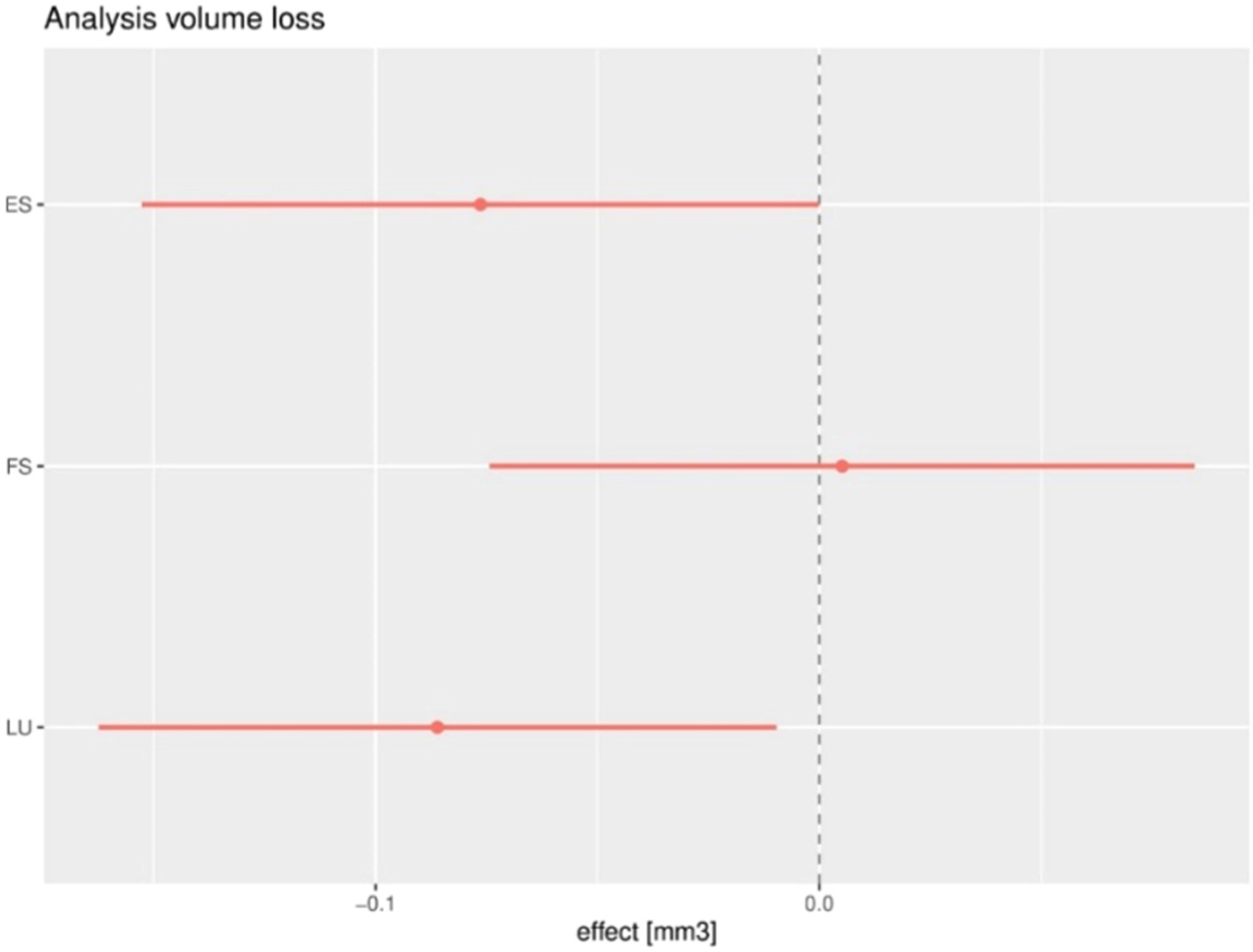
Results of the regression analysis. The 95 % CI of the wear volume of the regression analysis for ES, FS and LU are shown and compared to the reference group AP (0.0 N line).

**Fig. 6.**
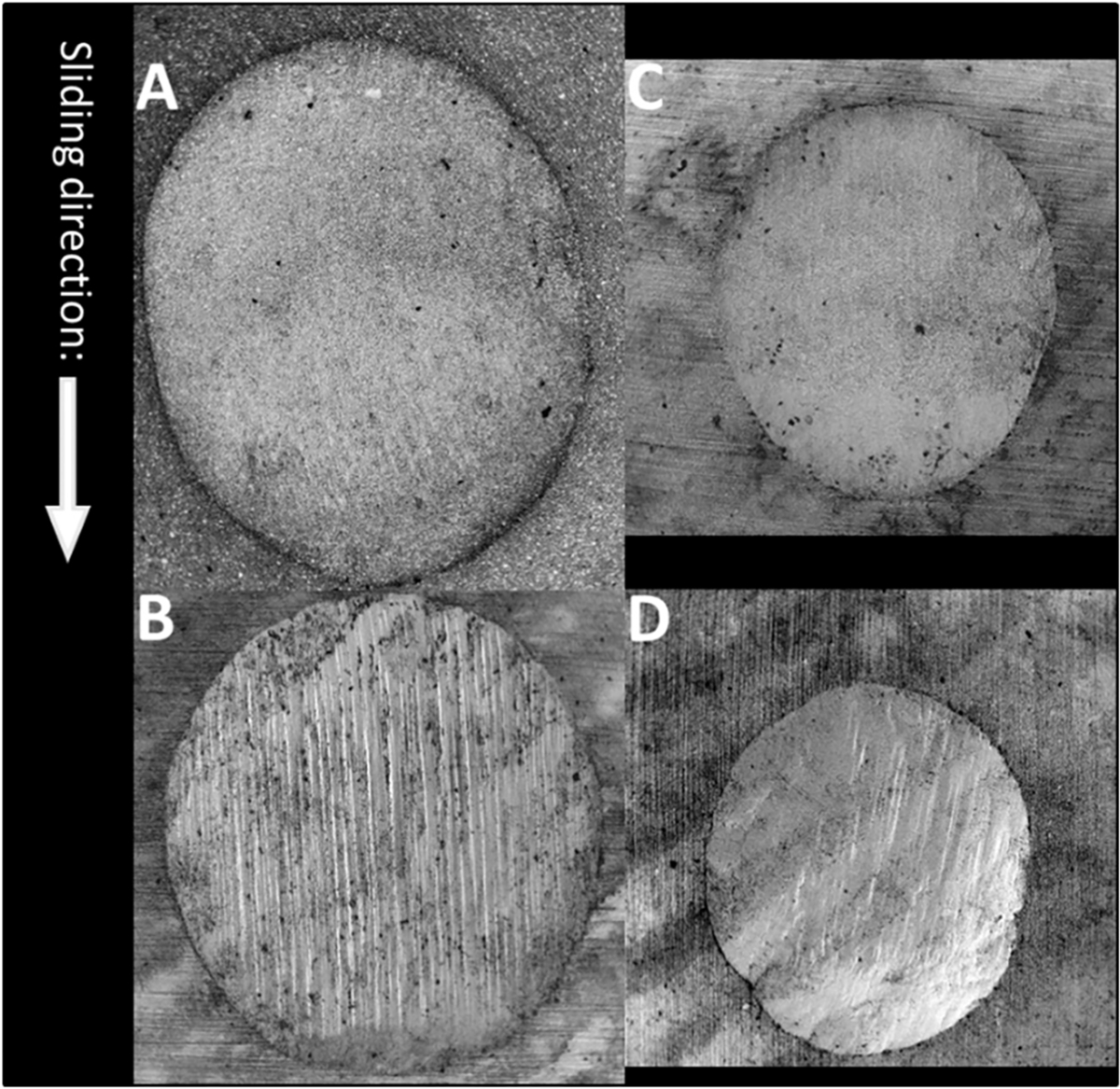
SEM images of examples of a worn surface the specimens. In the center of the images, an oval wear scar can be observed. These wear scars are a result of repeated contact-load-slide-liftoff motion (A: AP, B: FS, C: ES, D: LU).

The mean volume loss of material was 0.25 ± 0.09 mm^3^ for AP, 0.26 ± 0.08 mm^3^ for FS, 0.17 ± 0.05 mm^3^ for ES, and 0.16 ± 0.04 mm^3^ for LU. Results for the statistical comparison of wear volume of the four composite materials are graphically displayed in Fig. 5. With AP as a reference group, FS and ES showed a similar degree of volume loss, but LU had a significantly lower volume loss. Wear scars could be observed in all specimens that were subjected to the fatigue test. Typical examples of wear scars per material are displayed in Fig. 6.

**Fig. 7.**
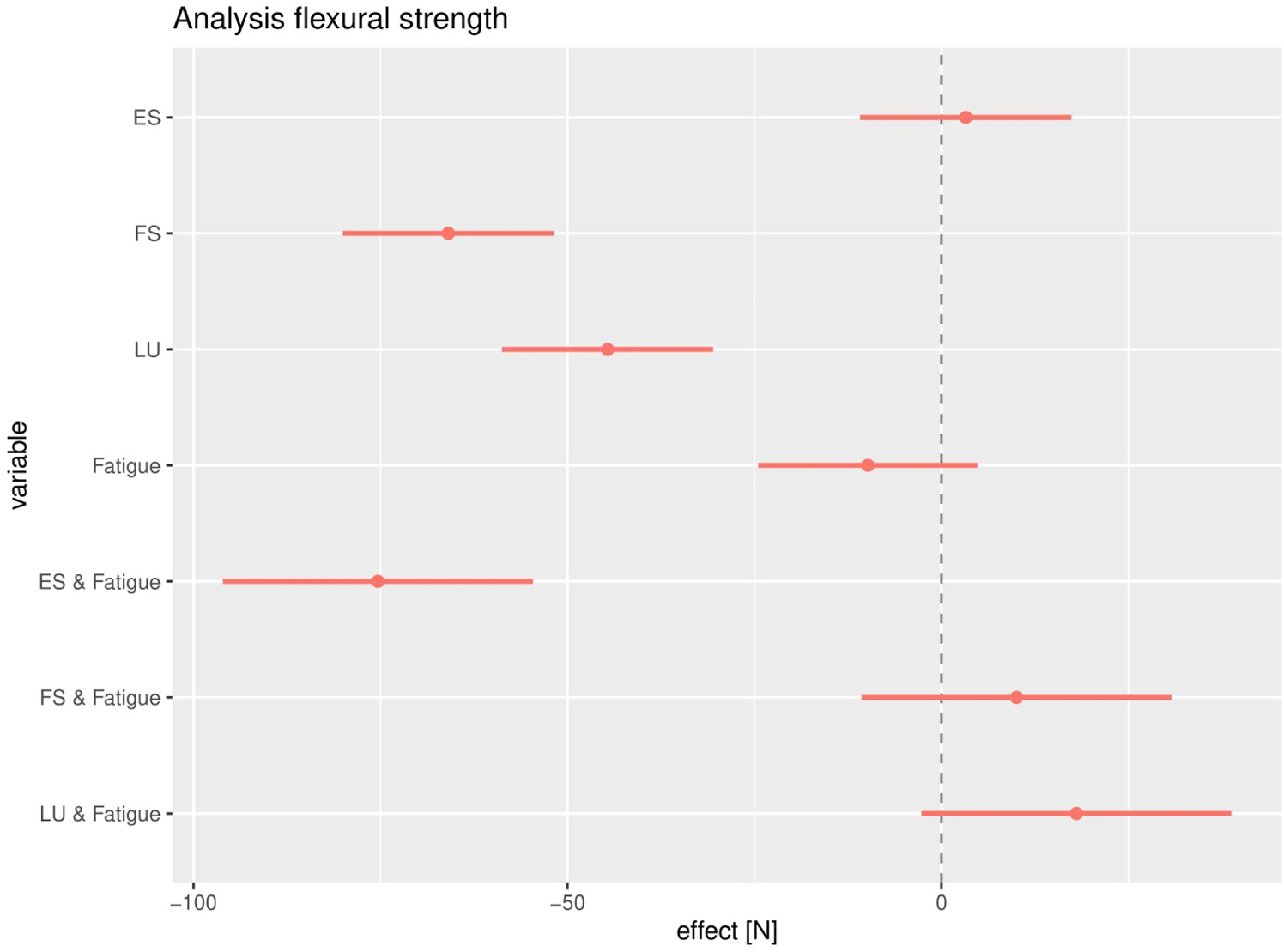
Graph of the 95 % CI of the flexural strength of the regression analysis. The dotted line on effect 0.0 N is the reference group which is non-fatigued AP. The intercept (dotted line on effect 0.0 N) can be regarded as AP specimens that did not receive the fatigue test. The effect of the material (without fatigue test) on the flexural strength is described for ES, FS, and LU, separately. The estimate (N) gives the mean change in flexural strength relative to the mean flexural strength of the intercept group (AP). ‘Fatigue’ refers to the effect of the fatigue test on the flexural strength. The ES/FS/LU + fatigue describes the effect of the fatigue test for each material on the flexural strengt.

The mean flexural strength for non-fatigued and fatigued specimens was 183 ± 12 and 174 ± 26 N for AP, 118 ± 18 and 118 ± 22 N for FS, 187 ± 20 and 102 ± 19 N for ES, and 139 ± 18 and 147 ± 18 N for LU, respectively. The flexural strength of the composite discs was analysed for the effect of the material, the effect of the fatigue test and the interaction between the material and the fatigue test. The 95 %CI of the mean values can be found in Figure 8, in which non-fatigued AP was used as the reference group. Non-fatigued ES showed similar flexural strength (p = 0.65; 95 %CI: −10.9 – 17.4). Both non-fatigued nanocomposites (FS + LU) had a significantly lower flexural strength compared to AP (p < 0.001; 95 %CI: −80.0 – 51.8), Fig. 7.

The fatigue test had no significant effect on the flexural strength of AP-specimens (p = 0.19; 95 %CI: −24.5 – 4.8). No significant effect of the interaction between the fatigue test and the material could be observed for FS and LU specimens (p > 0.09; −10.7 – 38.8). However, the interaction between the fatigue tests and the ES composite had a significantly negative effect on the flexural strength (p < 0.001; 95 %CI: −96.1 – −54.6).

## 4. Discussion

This study aimed to reproduce and translate clinical presentations in an *in vitro* set-up, evaluating laboratory outcomes of mechanical properties (flexural strength, fatigue resistance, and wear resistance) and linking them to the clinical outcomes of the employed materials in the RTWP. For wear, the indirect composite LU showed lower levels of volumetric loss due to the wearing-away in the fatigue test compared to the other materials. Another main outcome of this study was the higher initial biaxial flexural strength of hybrid composites (AP and ES) compared to the nanocomposites FS and LU. In addition, the ES composite material had an inferior fatigue resistance as ES specimens showed a significantly lower biaxial flexural strength for the fatigued specimens compared to the non-fatigued specimens, while other materials had similar flexural strength outcomes for fatigued and nonfatigued specimens.

The outcome of the clinical trial in the RTWP comparing direct (AP) and indirect composite (ES), showing many fractures of ES molar restorations (3), is according to the results of the present study, as ES showed a remarkable decrease in fracture strength in the fatigue test compared to AP. ES and AP specimens in the present study were made identically and it is likely that material properties of ES are responsible for the increased fracture rate found *in vivo*. Hence, other possible reasons for the inferior clinical results such as deficient dental lab work or application procedures by the dentist are less likely due to the clinical fractures of ES restorations (3). The outcome of the other study within the RTWP (4) showing comparable clinical success of indirect CAD/CAM restorations (LU) similar to direct AP restorations is also in accordance with the present study results and suggests again that the material properties of ES caused the fractures and not the difference in interface of bonded direct and cemented indirect restorations. As such, the present test may claim a certain level of clinical relevance as established clinical results are in accordance with lab results. Within that context, this *in vitro* test may also help to better explain fracture behaviour in clinical trials with often complex multivariate design by reducing the number of clinical necessary variables to solely the relevant materials. However, further research in this respect is necessary as, for example lab results not in accordance with clinical results for restorative materials, may either indicate a not-representative test design or the importance of other clinically variables, such as application procedures.

For severe tooth wear patients rehabilitated with restorations in increased vertical dimension, restoration fracture is the most often occurring clinical failure [3–5]. On the long-term, also wear of the restorative material may limit clinical success. The *in vivo* wear of micro-hybrid (AP) and nanocomposite (FS) restorations showed similar levels after 5 years of clinical service [7]. As clinical wear data of ES and LU are not yet available for comparison, we have to be careful in interpretation of clinical and lab wear data but it may well be that the present test design is also clinically relevant for wear behaviour, which will be subject of further investigation.

FS and LU are both nanocomposites and have similar organic and inorganic components with similar filler contents (w/v: 73/56 and 80/65), see Table 1. AP and ES are micro-hybrid composites with high inorganic filler content (w/v: 86/70 and 92/82) and higher Young’s moduli. The biaxial flexural strength of non-fatigued specimens was significantly higher for the hybrid composites compared to the nanocomposites. Higher filler contents of composites contribute to higher flexural strengths [26,27]. FS and LU are nanocomposites containing similar dispersed silica (20 nm) and zirconia (4–11 nm) nanoparticles and nanoclusters (0.6–10 μm) [13], and have lower filler contents and lower Young’s moduli compared to the hybrid composites. Zirconia has a higher hardness value than the other substances in the composite materials and steatite [28]. When the contact-load-slide-liftoff motion is repeatedly performed, filler clusters that contain zirconia particles may have dislodged. These dislodged particles may then have scratched the surface of the specimens in a form of abrasive wear. That may be a reason why scratches can be seen on the surface of the wear scar for FS and LU (Fig. 7).

The tested materials can be considered direct-indirect pairs of both micro-hybrid and nanocomposites. LU is delivered in industrially manufactured blocks, and ES, although it is delivered in unpolymerized form, comparable to a direct resin composite, is processed additionally with light curing and heat curing to increase mechanical properties. Keeping the similarities in mind, ES and LU may be roughly regarded as versions of respectively AP and FS with (expected) improved mechanical properties. Therefore, we would expect better outcomes on fracture strength, fatigue resistance, and wear resistance for these indirect composites compared to their ‘direct’ variant. Higher biaxial flexural strength of the CAD/CAM nanocomposite (LU) for both fatigued specimens and control specimens compared to the ‘direct’ variant (FS) was observed. Also, superior wear resistance was observed for LU compared to FS. These differences were, however, not significantly different, likely because of a lack of statistical power. The industrial process to manufacture the prepolymerized blocks may lead to increased mechanical properties due to maximum control of the quality and homogeneity of the material. No beneficial effect of the indirect processing (additional light and heat curing) on the mechanical properties could be observed for ES, when compared to its ‘direct’ variant AP. Similar flexural strength was found when no fatigue test was performed. Surprisingly, a significantly lower flexural strength for fatigued ES was observed while no reduction in flexural strength due to fatigue loading was observed for AP. Moreover, the lowest fracture resistance after cyclic loading was found for ES of all four materials. The reason for this is unknown. An explanation might be that the additional curing of ES may cause it to become brittle. Another explanation could be different water absorption behaviour of different composite materials as water uptake is known to deteriorate its mechanical properties.

To mechanically fatigue dental restorative materials, laboratory studies can use different laboratory set-ups to apply cyclic loading of a material [29–33]. Cyclic loading is often performed by an indenter that repeatedly applies a compressive load on the tested specimen. The specific configuration used in this study showed that wear and fatigue could be simultaneously employed for restorative materials as the slide motion induces damage by friction between the indenter and the material. In addition, the used configuration could induce subsurface cracks in ceramic and polymer-infiltrated ceramic network materials (PICNs), although no such subsurface cracks were observed for composite materials [18].

## 5. Conclusions

This study showed that the ES composite material had lower fatigue resistance compared to the other composite materials. The LU composite material had lower volumetric wear compared to the direct composite materials (FS + AP). Some of these findings concur with the outcomes of clinical studies on the longevity of these composite products in patients with tooth wear. The employed laboratory test may be regarded as clinically relevant since clinical presentations were successfully emulated in the laboratory.

## Supplementary Material

Supplementary Material

## Figures and Tables

**Table 1:** materials in the study including brand names, shades, Inorganic and organic characteristics, and mechanical properties conform the manufacturers.

	Manufacturer	Type	Content (w/v)	Organic Composition	Inorganic composition	Particle size	Compressive Strength (MPa) (according to manufacturer)	Young’s modulus (GPa) (according to manufacturer)	Flexural Strength (MPa) (according to manufacturer)

Cleaifil^™^ AP-X	Kuraray Medical, Osaka, Japan	Direct: Micro-hybrid composite	85.5/70	- Bis-GMA, - TEGDMA.	Silanated barium glass, Silanated silica, Silanated colloidal silica fillers.	between 0.1 μm and 15 μm (mean; 3 μm).	449	16.8	204
Filtek^™^ Supreme XTE	3 M ESPE Dental products, Seefeld, Germany	Direct: Nano-composite	72.5/55.6	-Bis-GMA, -TEGDMA, -UDMA, -Bis-EMA	Silica filler (20 nm). Zirconia filler (4–11 nm). Cluster of silica and zirconia fillers.	between 0.6 μm and 10 μm (average of cluster).	371	11.3	165
Esterna^™^ C&B	Kuraray Medical, Osaka, Japan	Indirect: Micro-hybrid composite	92/82	-BIS-GMA -UDMA -Hydrophobic aromatic dimethacrylate^2^-Hydrophobic aliphatic methacrylate^2^-Decandiol di methacrylate^3^.	Surface treated alumina (2 (μm) Silanated glass ceramics dl-Camphorquinone Initiators Accelerators Pigments^2^	0.02 to 2.0 μm	613	28.6	202
Lava Ultimate	3 M ESPE Dental products, Seefeld, Germany	Indirect:Nano-composite	80/65	Coupling agent: Silane.-Bis-GMA, -TEGDMA, -UDMA, -Bis-EMA	Silica filler (20 nm), zirconia filler (4–11 nm), Cluster of silica and zirconia fillers.	between 0.6 μm and 10 μm (average of cluster).	383	12.8	204

**Table 2–:** Regression analysis of the wear scar.

	Estimate (mm^3^)	95 % CI	P value

Intercept (AP)	0.25	0.194 – 0.302	
ES	−0.08	−0.153 – 0.000	0.05
FS	0.005	−0.074 – 0.085	0.89
LU	−0.09	−0.162 – −0.010	0.03 *

**Table 3–:** Regression analysis of the Flexural strength and the effect of the fatigue test.

	Estimate (N)	95 % CI	P value

Intercept (AP)	183.5	173.5 – 193.5	
ES	3.3	−10.9 – 17.4	0.65
FS	−65.9	−80.0 – 51.8	< 0.001 *
LU	−44.6	−58.8 – 30.5	< 0.001 *
The fatigue test	−9.8	−24.5 – 4.8	0.19
ES + fatigue test	−75.3	−96.1 – 54.6	< 0.001 *
FS + fatigue test	10.0	−10.7 – 30.8	0.34
LU + fatigue test	18.0	−2.7 – 38.8	0.09

The intercept can be regarded as AP specimens that did not receive the fatigue test. The effect of the material (without fatigue test) on the flexural strength is described for ES, FS, and LU, separately. The Estimate (N) gives the mean change in flexural strength relative to the mean flexural strength of the Intercept group (AP).

‘The fatigue test’ refers to the effect of the fatigue test on the flexural strength. The ES/FS/LU + fatigue test describes the effect of the fatigue test for each material on the flexural strength.

* = statistical significance was reached
