# Supplementary material for: An in vitro evaluation of the fatigue behavior of resin composite materials as part of a translational research cycle
Source: Dent Mater. Author manuscript; Available in PMC 2025 Sep 1. (PMC11330355; doi:10.1016/j.dental.2024.06.010)
Supplement: Supplementary Material [file NIHMS2006130-supplement-Supplementary_Material.docx]

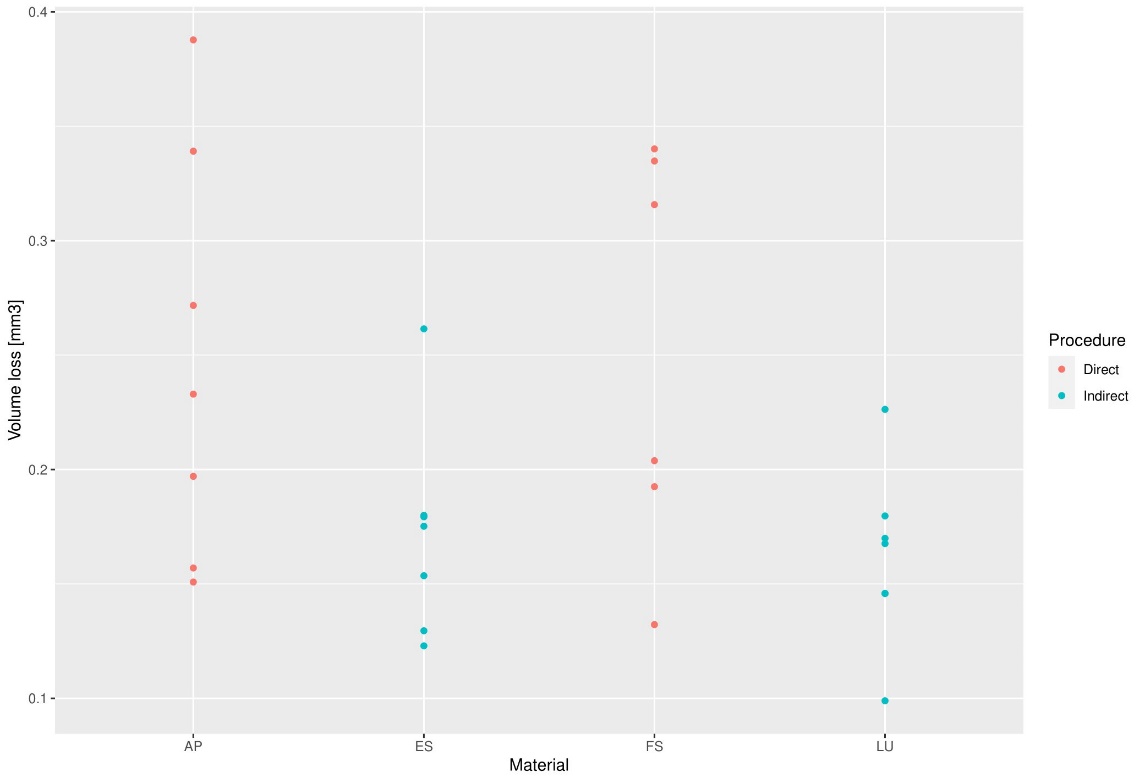
**Supplementary**


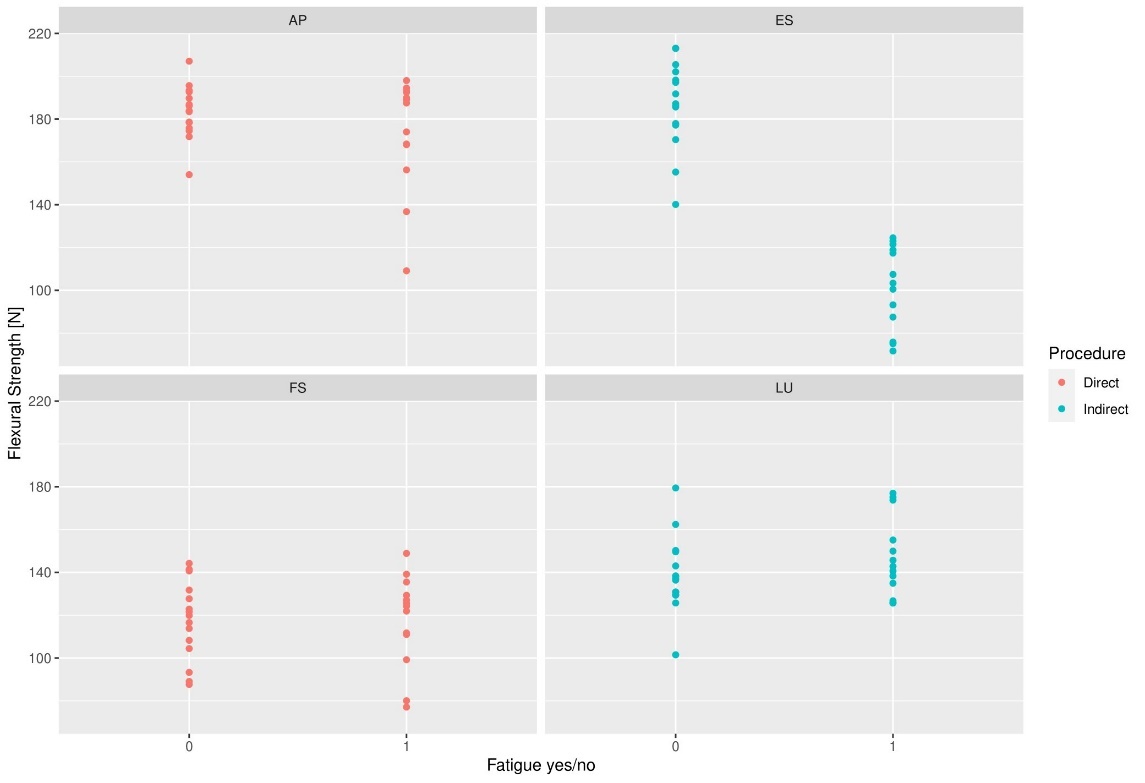


Figure 5: Descriptive graph of the flexural strength of the four composites separated for fatigued (1) and non-fatigued (0) specimens

Figure 8: Descriptive graph of the wear scar in volume loss of material for the four composites

| **Supplementary TABLE – Materials and Instruments used in the study** | |
| --- | --- |
| Materials | |
| Clearfil ^TM^ AP-X (Kuraray) | Direct micro hybrid composite |
| Estenia ^TM^ C&B (Kuraray) | Indirect micro hybrid composite |
| Filtek ^TM^ Supreme XTE (3M) | Direct nanofilled composite |
| Lava Ultimate (3M) | Indirect CAD/CAM composite |
| G10, Acculam (Yonkers) | Fiber glass-reinforced epoxy resin |
| Multilink® Automix (Ivoclar) | Resin cement |
| Monobond Plus (Ivoclar) | Silane coupling agent |
| IPS. Ceramic Etching Gel (Vita Zahnfabrik) | Hydrofluoric acid |
| Instruments | |
| Bluephase 16i (Ivoclar) | Light curing device |
| CEREC Primemill (Dentsply Sirona) | Milling machine |
| Ultra Lume LED 5 (Ultradent) | LED light curing device |
| ELF-3300, EnduraTEC Division (TA Instrument) | Mouth-motion stimlator |
| LAS-20, SD (Mechatronik GmbH) | Non-contact Laser Abrasion measurement System |
| Geomagic Wrap Software (v 2017) | Software programme |
| model 5566 (Instron) | Universal mechanical testing machine |
